# Progress in scale up of HIV viral load testing in select sub-Saharan African countries 2016–2018

**DOI:** 10.1371/journal.pone.0282652

**Published:** 2023-03-15

**Authors:** Peter N. Fonjungo, Shirley Lecher, Clement Zeh, Erin Rottinghaus, Helen Chun, Christiane Adje-Toure, Spencer Lloyd, Jane W. Mwangi, Michael Mwasekaga, Yohannes Mengistu Eshete, Rituparna Pati, Tsietso Mots’oane, Kiren Mitruka, Anita Beukes, Christina Mwangi, Nancy Bowen, Ndapewa Hamunime, Rachel S. Beard, Anyelwisye Kabuje, Susan Nabadda, Andrew F. Auld, Shirish Balachandra, Innocent Zungu, James Kandulu, George Alemnji, Eboi Ehui, Heather Alexander, Dennis Ellenberger

**Affiliations:** 1 Division of Global HIV and TB, Center for Global Health, CDC, Atlanta, Georgia, United States of America; 2 Division of Global HIV and TB, Center for Global Health, CDC, Abidjan, Côte d’Ivoire; 3 Division of Global HIV and TB, Center for Global Health, CDC, Nairobi, Kenya; 4 Division of Global HIV and TB, Center for Global Health, CDC, Dar es Salaam, Tanzania; 5 Division of Global HIV and TB, Center for Global Health, CDC, Maseru, Lesotho; 6 Laboratory Services, Ministry of Health, Maseru, Lesotho; 7 Namibia Institute of Pathology, Windhoek, Namibia; 8 Division of Global HIV and TB, Center for Global Health, CDC, Kampala, Uganda; 9 Ministry of Health, Nairobi, Kenya; 10 Ministry of Health and Social Services, Windhoek, Namibia; 11 Division of Global HIV and TB, Center for Global Health, CDC, Windhoek, Namibia; 12 Ministry of Health, Dar es Salaam, Tanzania; 13 Central Public Health Laboratories, Kampala, Uganda; 14 Division of Global HIV and TB, Center for Global Health, CDC, Lilongwe, Malawi; 15 Ministry of Health, Lilongwe, Malawi; 16 Office of the Global AIDS Coordinator and Health Diplomacy, U.S. Department of State, Washington, DC, United States of America; 17 Ministry of Health, Abidjan, Côte D’Ivoire; Chantal Biya International Reference Centre for HIV/AIDS Research on Prevention and Treatment: CIRCB, CAMEROON

## Abstract

**Introduction:**

We assessed progress in HIV viral load (VL) scale up across seven sub-Saharan African (SSA) countries and discussed challenges and strategies for improving VL coverage among patients on anti-retroviral therapy (ART).

**Methods:**

A retrospective review of VL testing was conducted in Côte d’Ivoire, Kenya, Lesotho, Malawi, Namibia, Tanzania, and Uganda from January 2016 through June 2018. Data were collected and included the cumulative number of ART patients, number of patients with ≥ 1 VL test result (within the preceding 12 months), the percent of VL test results indicating viral suppression, and the mean turnaround time for VL testing.

**Results:**

Between 2016 and 2018, the proportion of PLHIV on ART in all 7 countries increased (range 5.7%–50.2%). During the same time period, the cumulative number of patients with one or more VL test increased from 22,996 to 917,980. Overall, viral suppression rates exceeded 85% for all countries except for Côte d’Ivoire at 78% by June 2018. Reported turnaround times for VL testing results improved in 5 out of 7 countries by between 5.4 days and 27.5 days.

**Conclusions:**

These data demonstrate that remarkable progress has been made in the scale-up of HIV VL testing in the seven SSA countries.

## Introduction

Remarkable progress was made between 2013 and 2018 to curb the HIV epidemic. In 2013, 12.9 million people living with HIV (PLHIV) had access to ART worldwide [1]. By 2018, this number nearly doubled, rising to 23.3 million people worldwide [2]. As more patients were being put on life-saving treatment, the method for diagnosing and monitoring treatment failure also evolved. Prior to June 2013, the primary method used to monitor treatment effectiveness in sub-Saharan Africa (SSA) was CD4 cell count, with targeted HIV viral load (VL) testing only being used for suspected treatment failures. In June 2013, the World Health Organization (WHO) recommended VL testing as the preferred test to monitor patients on ART, resulting in a slow, yet drastic change in the HIV laboratory diagnostic landscape [3]. VL monitoring is not only critical for the measurement of ART effectiveness and clinical management of patients, but also allows the measurement of country ART programs’ progress towards the third 95 of the Joint United Nations Programme on HIV/AIDS (UNAIDS) 95-95-95 Fast-track targets by 2030 [4]. UNAIDS 95-95-95 targets are defined as 95% of people living with HIV know their status, and 95% of all diagnosed PLHIV are on anti-retroviral treatment, and 95% of those on treatment having viral load suppression (defined as < 1,000 cps/mL) [4]. VL testing serves as an important tool to implement patient-centered differentiated care so that patients who are virally suppressed may be seen less often by their health care providers, while those not suppressed are targeted for enhanced adherence counselling and closer monitoring for the potential switch to second-line ART.

The implementation of the 2013 WHO recommendations for the use of VL as the preferred monitoring test for patients on ART presented substantial challenges for countries in SSA with limited resources. At the beginning of VL scale-up, multiple SSA countries had limited molecular diagnostic capabilities, primarily due to high operational costs. Several factors played a role in the successful implementation and scale-up of VL testing in resource-limited settings at this time. First, effective global partnerships have been critical, involving key stakeholders such as the U.S. President’s Emergency Plan for AIDS Relief (PEPFAR), UNAIDS, the World Health Organization (WHO), the Global Fund to Fight AIDS, Tuberculosis and Malaria (GFATM), the Clinton HIV Access Initiative (CHAI), United Nations Children’s Fund (UNICEF), and others. Contributions of these stakeholders include sustained advocacy, funding, and technical assistance, including development and dissemination of tools to facilitate VL scale-up [5, 6]. Second, Ministries of Health (MOH) have facilitated effective coordination among multiple partners, adopted evidence-based policies, and fostered an enabling environment for continuous quality improvement (CQI) and more accurate forecasting for reagents and commodities [7]. Third, concerted efforts by donors and stakeholders have significantly reduced costs of VL reagents and accelerated reagent bundling to address equipment maintenance issues and reduce interruption in VL testing services [8]. Fourth, technologic advancements by manufacturers have improved access to VL testing through higher efficiency and throughput testing platforms [9]; the use of alternative specimen types such as dried blood spots [10], plasma separation cards [11], and point of care technology [12, 13] have expedited results return for timely clinical management. Fifth, HIV programs, implementing partners, and civil society organizations have made strong efforts in client demand creation, community mobilization, and facility-level systems’ strengthening to improve access to and utilization of VL testing and results. Lastly, donors, stakeholders and implementing partners have addressed national and site-level systems’ challenges, committing to steady progress in laboratory CQI and accreditation [14–17]. Finally, the 2016 WHO recommendations for universal access to ART for all PLHIV irrespective of CD4 results prompted countries to improve infrastructure, and increase the capacity of laboratories, and health care facilities to respond to the increase demand for ART and VL testing [18].

PEPFAR’s support of national programs together with stakeholders and other donors has been critical for millions of individuals to know their HIV status, initiate ART, and receive treatment monitoring to measure success towards achieving HIV epidemic control [19–23]. Ministries of Health have been at the forefront of the rapid expansion of case identification, treatment initiation and monitoring through the swift adoption of evidence-based policies such as ‘test and start” and use of VL testing as the preferred method for monitoring of patients on ART [18].

The aim of this study was to assess progress in scale-up of VL testing in seven countries in SSA between 2016 and 2018, and to discuss barriers as well as solutions for improvements.

## Materials and methods

### Program description

This study assessed the progress of VL scale-up in seven SSA countries (Côte d’Ivoire, Kenya, Lesotho, Malawi, Namibia, Tanzania, and Uganda) between January 2016 and June 2018. These countries were selected based on their willingness and agreements with the MOH of each country to participate in this study and availability of reported data. Six of the seven countries have adopted WHO recommendations for VL monitoring at 6 and 12 post-ART initiation and every 12 months thereafter for stable patients. In Malawi, VL monitoring was conducted every 24 months for stable patients, until the policy changed in 2019 to WHO standard care [18, 24].

### Data collection and analyses

A questionnaire was designed and used for data collection from multiple sources including laboratory information systems, laboratory and clinic registers, and/or MOH national dashboards. Data from each of the 7 countries were collected between January 2016 and June 2018. MOH and CDC program officers jointly collected information on cumulative number of ART patients (adults and children), the number of ART patients with ≥ 1 VL test result, and the mean turnaround time (TAT). The mean TAT was defined as the time (in days) from sample collection to the time VL results were returned from the testing laboratory to the collecting facility. The number of results with viral suppression was calculated by dividing the number of viral load tests with <1000 copies/mL by total number of VL test results. Data on the number of PLHIV were obtained from the UNAIDS database [25] and used to estimate the proportion of PLHIV accessing ART (i.e., ART coverage). Data on the number of PLHIV on ART and routine VL tests from 2014 were used as a baseline [21] to estimate percentage increases when compared to the last full year of data from 2017 (only 6 months of 2018 were available). Information related to CQI efforts, activities aimed at strengthening laboratory management towards accreditation [26], and external proficiency testing were also obtained for all VL testing laboratories.

The percentage of VL tests with viral suppression and the mean TAT for return of VL results were calculated and compared across countries.

### Ethical clearance

The study protocol was reviewed in accordance with the U.S. Centers for Disease Control and Prevention (CDC) human research protection procedures and was determined to be non-research. Applicable federal law for ethical review include: 45 C.F.R. part 46.102(l)(2), 21 C.F.R. part 56; 42 U.S.C. §241(d); 5 U.S.C. §552a; 44 U.S.C. §3501 et seq. Ethical clearance on this non-research project was also received from the Ministry of Health from Cote d’ Ivoire, Kenya, Lesotho, Malawi, Namibia, Tanzania, and Uganda.

## Results

### PLHIV, ART patients, and VL testing

All 7 countries had VL testing laboratory networks that supported the goal of increasing VL testing coverage of patients on ART. Côte d’Ivoire, Kenya, Lesotho, Malawi, Namibia, Tanzania and Uganda had 14, 8, 5, 10, 8, 16 and 1 laboratories, respectively, that supported specific clinical sites, with the goal of establishing quality laboratory services and efficient sample transport networks. All the laboratories in these 7 countries participated in continuous quality improvement activities and were either working towards or received international accreditation (data not shown).

The number of PLHIV and ART patients in the seven countries markedly increased between January 2016–June 2018, increasing the demand for VL testing. Kenya had the greatest number of ART patients with 969,433 (approximately 64.6% of PLHIV) and 1,069,451 (66.8% of PLHIV) in 2016 and 2018, respectively. The smallest volume of ART patients was in Lesotho (126,706; 37.3% of PLHIV) in 2016, and in Namibia (171,014; 85.5% of PLHIV) in 2018 (Table 1). Lesotho experienced the largest increase (50.2%) in the ART patient volume, while Namibia showed the smallest increase of 5.7% over the 30-month period. Growth in the remaining countries’ ART patient volume ranged from 10.3% to 34% as follows: Kenya (10.3%), Malawi (25.3%), Côte d’Ivoire (28.1%), Uganda (18.1%) and Tanzania (34.0%). In 2018, ART coverage among PLHIV per country ranged from 49.9% (Côte d’Ivoire) to 85.5% (Namibia). ART coverage in the other countries was Lesotho (56%), Tanzania (62.5%), Kenya (66.8%), Uganda (73.7%), and Malawi (74.6%). Over this 30-month period, all the countries experienced increases in ART coverage: Kenya (3.4%), Namibia (5.7%), Uganda (9.7%), Malawi (25.3%), Tanzania (25.6%), Côte d’Ivoire (30.9%) and Lesotho (50.2%). The 3 countries experiencing the smallest increase in ART coverage over the 30- month period were Kenya, Namibia and Uganda but they were the countries with the highest ART coverage in 2016.

**Table 1:** Select VL monitoring indicators from 2016–mid-year 2018 by country.

Country	# PLHIV^a^	# ART patients^b^	% ART coverage	# of patients with ≥1 VL test ^c^	% of VL tests with VS^d^	#PLHIV	# ART patients	% ART coverage	# of patients with ≥1 VL test ^c^	% of VL tests with VS^d^	# PLHIV	# ART patients	% ART coverage	# of patients with ≥1 VL test ^c^	% of VL tests with VS^d^
	2016					2017					Jan-Jun 2018				
Cote d’Ivoire	470,000	179,045	38.1	49,341	76.0	460,000	221,990	48.3	134,849	77.0	460,000	229,399	49.9	92,781	78.0
Kenya	1,500,000	969,433	64.6	725,339	81.0	1,500,000	1,041,326	69.4	861,439	86.0	1,600,000	1,069,451	66.8	917,980	88.0
Lesotho	340,000	126,706	37.3	22,996	87.0	340,000	151,799	44.6	66,296	90.0	340,000	190,375	56.0	72,759	90.0
Malawi	1,000,000	595,186	59.5	276,673	87.7	1,000,000	679,056	67.9	310,701	86.9	1,000,000	745,532	74.6	170,491	85.0
Namibia	200,000	161,716	80.9	182,120	86.5	200,000	167,629	83.8	214,111	87.0	200,000	171,014	85.5	112,252	88.4
Tanzania	1,500,000	746,005	49.7	96,320	79.0	1,500,000	932,466	62.2	330,476	84.0	1,600,000	999,628	62.5	499,795	85.0
Uganda	1,300,000	874,124	67.2	664,687	88.9	1,400,000	1,012,867	72.3	943,523	88.0	1,400,000	1,032,383	73.7	503,679	87.8
Total	6,310,000	3,652,215		2,017,476		6,400,000	4,207,133		2,861,395		6,600,000	4,437,782		2,369,737	

^a^ Number of people living with HIV [25]

^b^ Number of adult and pediatric patients currently receiving antiretroviral therapy (ART).

^c^ Number of adult and pediatric patients currently on ART with ≥1 VL test.

^d^ Percentage of VL test results with <1,000 copies/mL.

In 2016, the number of ART patients with one or more VL tests ranged from 22,996 (Lesotho) to 725,339 (Kenya). Comparing 2017 to 2016, Malawi, Namibia and Kenya demonstrated 12.3%, 17.6%, and 18.8% increases, respectively, in ART patients with one or more VL test. Increases of 41.9%, 173.3%, 188.3% and 243.1% were observed for Uganda, Côte d’Ivoire, Lesotho and Tanzania, respectively (Table 1). By the end of June 2018, the number of ART patients with one or more VL tests ranged from 72,759 (Lesotho) to 917,980 (Kenya). Among countries initiating routine VL monitoring scale-up in 2014 to the end of 2017 (last full year), the proportion of ART patients with VL test results increased from 60.5% to 127.7% in Namibia, 4.9 to 93.2% in Uganda, 8.4% to 82.7% in Kenya, 3.8% to 60.7% in Côte d’Ivoire, 6% to 45.8% in Malawi, and 2.4% to 35.4% in Tanzania. Similar to the rise in the number of patients on ART for all countries, the number of patients on ART receiving one or more VL tests increased across all countries (Table 1).

### VL suppression

In 2016, the proportion of VL tests demonstrating viral suppression was greater than 85% in 4 (Namibia, Malawi, Lesotho and Uganda) of the 7 countries, ranging from 86.5% (Kenya) to 88.9% (Uganda) (Table 1). Côte d’Ivoire (76%), Tanzania (79%), and Kenya (81%) had viral suppression rates less than 85%. Tanzania and Kenya increased to 85% and 88% viral suppression by June 2018, respectively, and Côte d’Ivoire increased to 78%. By mid-2018, 6 of the 7 countries reported viral suppression rates greater than 85%. Lesotho reported the highest viral suppression at 90%. From 2016 to 2018, viral suppression rates increased the most in Kenya (8.6%) and Tanzania (7.6%) while decreasing in Malawi and Uganda by 3.1% and 1.2%, respectively, with modest increases in Namibia (2.2%), Côte d’Ivoire (2.6%) and Lesotho (3.4%).

### Turnaround time for VL testing

**Fig 1:**
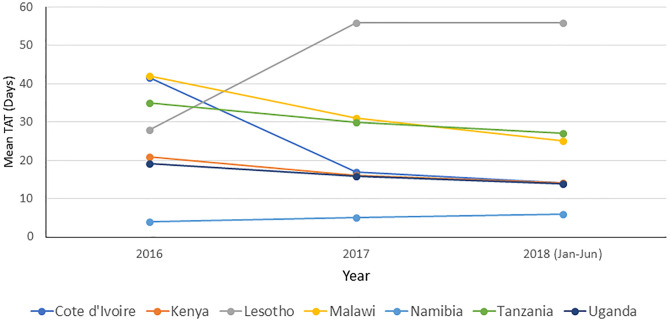
Turnaround time for viral load specimens in seven select countries, 2016–2018. Mean TAT in days (specimen collection to result dispatch) for all VL results returned during the monitoring period.

In 2016, the mean TAT from specimen collection to return of VL results to the referring clinic ranged from 4 days (Namibia) to 42 and 41.5 days in Malawi and Côte d’Ivoire, respectively (Fig 1). By June 2018, 5 of the 7 countries improved their TAT by 5.4 days (Uganda, 19.2 to 13.8 days), 7 days (Kenya, 21 to 14 days), 8 days (Tanzania, 35 to 27 days), 17 days (Malawi, 42 to 25 days) and 27.5 days (Côte d’Ivoire, 41.5 to 14 days)—Fig 1. In contrast, there was a 2-fold increase in TAT in Lesotho from 28 to 56 days and increased by 50% in Namibia from 4 to 6 days.

## Discussion

The 2016 WHO recommendations for universal access to ART for all PLHIV prompted countries to improve their infrastructure, and increase the capacity of laboratories and health care facilities to respond to the increase in demand for ART and VL testing [18]. From January 2016 to June 2018, all 7 SSA countries in this report made significant progress in the scale-up of VL testing as the number of PLHIV receiving ART increased. Lesotho, a small country in Southern Africa with the lowest number of patients on ART in 2016, experienced the largest percent increase in ART coverage (50.2%). Dramatic increases in the number of patients receiving VL tests from 2016 to 2017 for countries such as Côte d’Ivoire, Lesotho and Tanzania (173.3%, 188.3% and 243.1%, respectively) demonstrated the ability of low-income countries to rapidly scale up VL testing.

By mid-year 2018 all countries except Côte d’Ivoire achieved >85% VL suppression rates for patients on ART who received a VL test, suggesting successful treatment outcomes for the majority of PLHIV on ART. Of the 7 countries, 5 completed Population-based HIV/AIDS Impact Assessments surveys (Namibia, Tanzania, Uganda, Malawi and Lesotho) during November 2015 to December 2017 [27] and were found to have high viral suppression rates, greater than 84%, similar to our results for 2018 [28]. The reasons for a lower viral suppression rate observed in Côte d’Ivoire (76%) as compared with the other 6 countries in our study is unclear, but may be related to factors such as the following: treatment interruption (potentially driven by patient factors or facility-level stockouts), use of non-optimized ART regimens, slower implementation of differentiated service delivery, and lower VL testing coverage and consequently suboptimal patient management, and/or potential viral diversity associated factors leading to lower viral suppression [29, 30].

TAT for VL results is critical for the timely management of patients. TAT improved for most countries between January 2016 and June 2018. Despite TAT improvements, there are still opportunities to optimize VL testing networks and improve the laboratory-clinic interface. Routine extended TATs of ≥ 2 weeks suggest additional strengthening of specimen transport systems may be necessary. Facilities lacking electronic results and relying on the return of paper based VL results generally face challenges with the timely return of results to patients. Lesotho was the only country observed to have a decline in TAT by > 2 weeks from 28 days in 2016 to 56 days in over the study period. The reasons for the delayed TAT in Lesotho include intermittent backlogs of VL testing due to frequent equipment breakdown, and expansion of sample collection from remote areas often leading to batching prior to VL testing. These problems highlight persistent weaknesses in VL testing networks and service delivery within the VL cascade. Focused attention and effort is needed for continuous TAT improvement through the review and streamlining of laboratory and clinical workflows, and resolving challenges with specimen types (DBS and plasma) bearing in mind the distance and time to centralized laboratory testing [31]. Furthermore, strategic introduction of point of care VL testing to reduce TAT by providing same day results may be considered as has been demonstrated in Zimbabwe [32].

There are several limitations with our study. First, it is unclear if less than optimal viral suppression may be related to targeted VL load testing for suspected treatment failures as opposed to routine VL testing. Among VL results, we were not able to distinguish those reflecting repeat testing for patients who had already achieved VL suppression. Demographic information of patients on ART was limited, and we were unable to disaggregate VL suppression data by gender and age. While the data template requested that VL test results reported from the laboratory information system be de-duplicated by patient, we were unable to ensure that de-duplication occurred due to known gaps in unique patient identifiers and challenges obtaining patient-level data in SSA countries. Despite the challenges, there has been great progress in VL scale-up across the 7 SSA countries assessed in this study.

The knowledge and experience gained since the inception of VL testing have been critical in the identification of specific challenges at both health facility site and national levels. To accelerate the efficient scale-up of VL testing, sample transport systems may be strengthened, especially in rural and hard-to-reach areas [33]. Specific areas of focus within laboratory systems requiring greater attention include use of unique patient identifiers, forecasting of reagents and commodities, prevention of testing backlogs, retention of trained personnel, quality control, the laboratory-clinic interface, TAT, and data performance review and utilization for improvement. Barriers to VL scale-up at health facilities include low demand by clients and clinicians for VL testing, incomplete VL requisition forms leading to potential specimen rejection, and limited use of analyses disaggregated by age and sex to identify gaps, and poor utilization of VL results for patient management [34, 35]. Across the VL cascade there is a need for the collection of accurate data and data utilization to inform targeted interventions for improvement. Strategic placement of point of care VL testing capacity within the laboratory diagnostic network to increase access in hard-to-reach areas may help to improve VL testing coverage and reduce TAT amongst priority populations such as pregnant and breastfeeding women and children [36]. There may be a need for strong partnerships between manufacturers and in country procuring agencies and testing laboratories for planning and early projections. In addition, manufacturers should consider responding promptly and timely resolving equipment breakdowns while assuring timely delivery of orders of supplies and reagents to avoid testing backlogs and interruptions in service delivery. This can also be achieved through diagnostic network optimization that has been implemented in some countries and partly responsible for some of the impactful viral load scale-up results obtained [37].

In conclusion, as countries strive to reach the 95-95-95 fast track UNAIDS targets by 2030, there will be an increase in HIV diagnoses and individuals accessing optimized ART, requirements for routine VL monitoring and need for efficient systems to support sustained community viral suppression and the goals of achieving epidemic control [38]. Innovative strategies; the ongoing commitment of government, global partners and industry; and a collaborative, multidisciplinary approach involving clients, civil society, community-based organizations, laboratorians and clinicians remain critical to these efforts.
